# Seed biologists beware: Estimates of initial viability based on ungerminated seeds at the end of an experiment may be error‐prone

**DOI:** 10.1111/plb.13407

**Published:** 2022-03-02

**Authors:** B. B. Lamont

**Affiliations:** ^1^ Department of Molecular and Life Sciences (Ecology Section) Curtin University Perth WA Australia

**Keywords:** Experimental conditions, germination, seed viability estimation, seed viability loss

## Abstract

Seed viability is routinely measured on seeds that fail to germinate at the end of an experiment. Together with the number of germinants, this is used to estimate viability of the seeds at start of the experiment (i.e., initial viability) and provides the comparative basis on which germination success is determined. The literature and recent data on the germination requirements of Leucadendron species were examined to determine if there was any evidence for a treatment effect on viability of ungerminated seeds at the end of the experiment. The survey showed that sometimes (perhaps often, as the problem has yet to be recognized or reported) prolonged duration in the treatment, especially the control where little germination occurs, lead to loss of viability during the experiment. This resulted in underestimation of initial viability if that treatment was used. I caution against the routine use of end‐of‐trial germination and viability of ungerminated seeds as an estimate of initial viability in determining germination success of various treatments. I explore ways to deal with the problem but the preference is for estimates of initial viability to be undertaken on a separate sample of seeds concurrently with the experiment as this avoids the risk of seed death during the trial.

## Graphical abstract

It is recommended to determine initial seed viability at the start of the experiment, since prolonged duration of ungerminated seeds in germination treatments can lead to loss of viability.

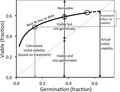

## THE PROBLEM

Determining initial viability is a vital part of testing the germination potential and requirements of seeds, since germination success can only be compared against seeds that were viable and therefore capable of germinating. Researchers usually estimate seed viability at the start of a trial/experiment (initial viability) on the condition of seeds remaining at the end of the trial plus those that had germinated. A representative sample of 13 of mostly recent studies are described in Table 1, which also serve to highlight the great range of methods used to estimate viability, but all used viability at the end to estimate viability at the start of the trial. Thus, estimated initial viability as a fraction is given by: (viable seeds present at end of trial + germinants)/(total seeds in trial). The results are then corrected for viability: (seeds germinated)/(total seeds in trial × initial viability), as described by Gosling (2003).

**Table 1:** Examples of studies that used assessment of seed viability and germination at the end of the experiment to estimate initial viability.

species tested	method of determining initial viability from end‐of‐experiment data	experimental conditions	differing initial viability between treatments	reference
21 woody native species of Chilean matorral	Viability calculated as: those germinated during the monitoring period plus non‐germinated seeds identified as viable by tetrazolium test	Moist absorbent paper in Petri dishes at 20/10 °C with 12/12 h light/dark for 36 days	Yes (3 species), but data not adjusted for viability	Gómez‐González *et al*. (2017)
13 native and 1 introduced species (*Acacia saligna*) of South African fynbos	Viability determined as the sum of germinated seeds and seeds appearing fresh on dissection of ungerminated seeds	1% water agar at 10/20 °C with a 12/12 h light/dark cycle for 91 days	Yes (at least 2 species)	Hall *et al*. (2017)
65 species commonly occurring on New England tableland (NSW Australia)	Seeds that did not germinate, but looked viable, were analysed using tetrazolium test. Viability based on treatment with highest germination plus any seeds that remained dormant but viable	Moist pad in dish at 25/15 °C with a 12/12 h light/dark for 28 or 56 days	Probably (as used highest viability levels between treatments)	Clarke *et al*. (2000)
*Asterolasia buxifolia,* riparian habitat of SE Australia	Embryo dissected from 20 seeds that did not germinate and viability confirmed if embryo and endosperm intact	Moist filter paper in Petri dishes at 11/3 °C with 12/12 h light/dark for 77 days	Data not adjusted for viability^a^	Collette & Ooi (2017)
33 herb and small shrub species in fire‐prone Turkey	Embryo of seeds that did not germinate examined and viability confirmed if embryo intact	0.8% agar in Petri dishes at 20 °C in dark for 35 days	Data adjusted for viability	Serter Çatav *et al*. (2018)
46 legumes species of tropical savanna, Brazil	Initial viability equals the sum of germinated and dormant seeds in control	Moist filter paper in Petri dishes at 27 °C with 12/12 h light/dark for 28 days	No data given at treatment level	Daibes *et al*. (2019)
13 species of West African savanna woodland	Cut test – condition of embryo, conducted on ungerminated seeds post‐trial	Moist filter paper in bell jars at 25 °C light for 30 days	No data given at treatment level	Dayamba *et al*. (2008)
9 species in Brazilian Cerrado	Cut test for post‐treatment seed viability	0.9% water agar in Petri dishes at 25 °C with a 12/12 h light/dark for 30 days	No	Fernandes *et al*. (2020)
7 native perennial forb species of grasslands and woodlands of SE Australia	Cut test – condition of embryo on ungerminated seeds post‐trial	1% water agar at 25/15°C with a 12/12 h light/dark for 56 days	No data given at treatment level	Hodges *et al*. (2019)
2 alien and 2 indigenous legume species in South African fynbos	Germination level of scarified seeds conducted at same time as other treatments	0.02% benomyl solution in Petri dishes at 20 °C with 12/12 h light/dark for 30 or 60 days	No data given at treatment level	Jeffrey *et al*. (1988)
3 species of *Acronychia* in E Australian rainforest	Ungerminated seeds checked for firmness by pressing seed with forceps. Then firm seeds checked for viability via cut test	0.8% water agar in Petri dishes at 25/10 °C with a 12/12 h light/dark for 28 days	No data given at treatment level	Liyanage *et al*. (2020)
9 herbaceous species in Brazilian grassland	Tetrazolium test on ungerminated seeds	Moist filter paper in Petri dishes at 20 °C or 25 °C at 16/8 h light/dark for 21 days	No data given at treatment level	Overbeck *et al*. (2006)
*Brassica napus*	Cotyledon condition of ungerminated seeds post‐trial. Necrotic cotyledons = nonviable; yellow‐milky cotyledons = viable	Moist filter paper in Petri dishes at 20 °C in dark for 35 days	No	Shayanfar *et al*. (2020)

^a^ ’Initial’ viability 80% at 100 °C but only 65% at the lower temperature of 80 °C appears anomalous and might indicate an unexpected treatment effect on viability (they should have been the same or the reverse if there was a eat effect on viability), but no statistical analyses were undertaken.

This standard procedure economizes on the number of seeds needed for the trial as it is not necessary to ‘waste’ seeds by testing for viability on separate samples before the trial begins. This can be important when seeds are scarce if the species is rare, or seeds are difficult to collect or expensive to purchase. It also removes any time‐lapse loss of viability between estimating viability before the trial begins and undertaking the trial itself. Further, it expedites the testing task as only ungerminated seeds need be examined for their viability. It also avoids the need to use a mean value obtained pre‐trial to apply to all treatments, which ignores sample effects on the viability of seeds used, as ‘actual’ initial viability of the seeds used in each replicate can be determined.

During a study of the germination requirements of *Leucadendron* species in relation to alternating temperatures, smoke and heat (Newton *et al*. 2021), I noticed that estimated initial viability using this standard procedure varied greatly between some treatments in a number of species when they should have been at least non‐significantly different. Estimated viability declined the lower the level of germination, *i.e*. usually among the controls. This indicated that there might be an unanticipated treatment effect on the viability of ungerminated seeds at the end of the trial.

Our solution at the time was to abandon this method used routinely in estimating viability of species at the Millennium Seed Bank (inspection of the condition of the embryo by ‘cut’ test when seeds are tested for their ability to remain viable during cold storage; Hall *et al*. 2017). Instead, those seeds that had not experienced microbial infection during the trial were treated as the seeds that were initially viable. This solution proved to be unaffected by the treatment, *i.e*. infected seeds had indeed been randomly allocated to the various treatments. However, it is possible that some of the remaining seeds were still nonviable, although only the cut test applied at the *start* of the trial would have addressed this. In fact, the large number of species with 100% viability by the ‘uninfected’ method implies that initial viability may indeed have been overestimated.

I checked the literature and found that Hay & Probert (2013) had warned that seeds kept for prolonged periods under (apparently suboptimal) experimental conditions can die, although they did not provide any supporting data or references. Inspection of the 13 representative papers that used the end‐of‐trial approach described in Table 1 revealed several with variable post‐trial estimates of initial viability that were not commented on by the authors. That is, estimates of initial viability varied markedly between treatments and, unexpectedly, were especially low among the untreated controls. This included Hall *et al*. (2017), working on species in the South African heathlands, and Gómez‐González *et al*. (2017), working on shrub species in central Chile.

Clarke *et al*. (2000), working on species in grassy eucalypt woodland in Australia, might also provide another example, as this would explain why they chose to use the treatment result that gave the highest estimate of initial viability. For this begs the question: why was there a difference in estimated initial viability between treatments that required a choice to be made? Was it just random error (in which case the correct solution would be to take the mean) or was it systemic? Of additional concern is that nine of the papers either did not report estimates of initial viability on a per treatment basis (so I could not assess whether this was a problem or not) or did not adjust for it in determining germination success.

**Fig. 1:**
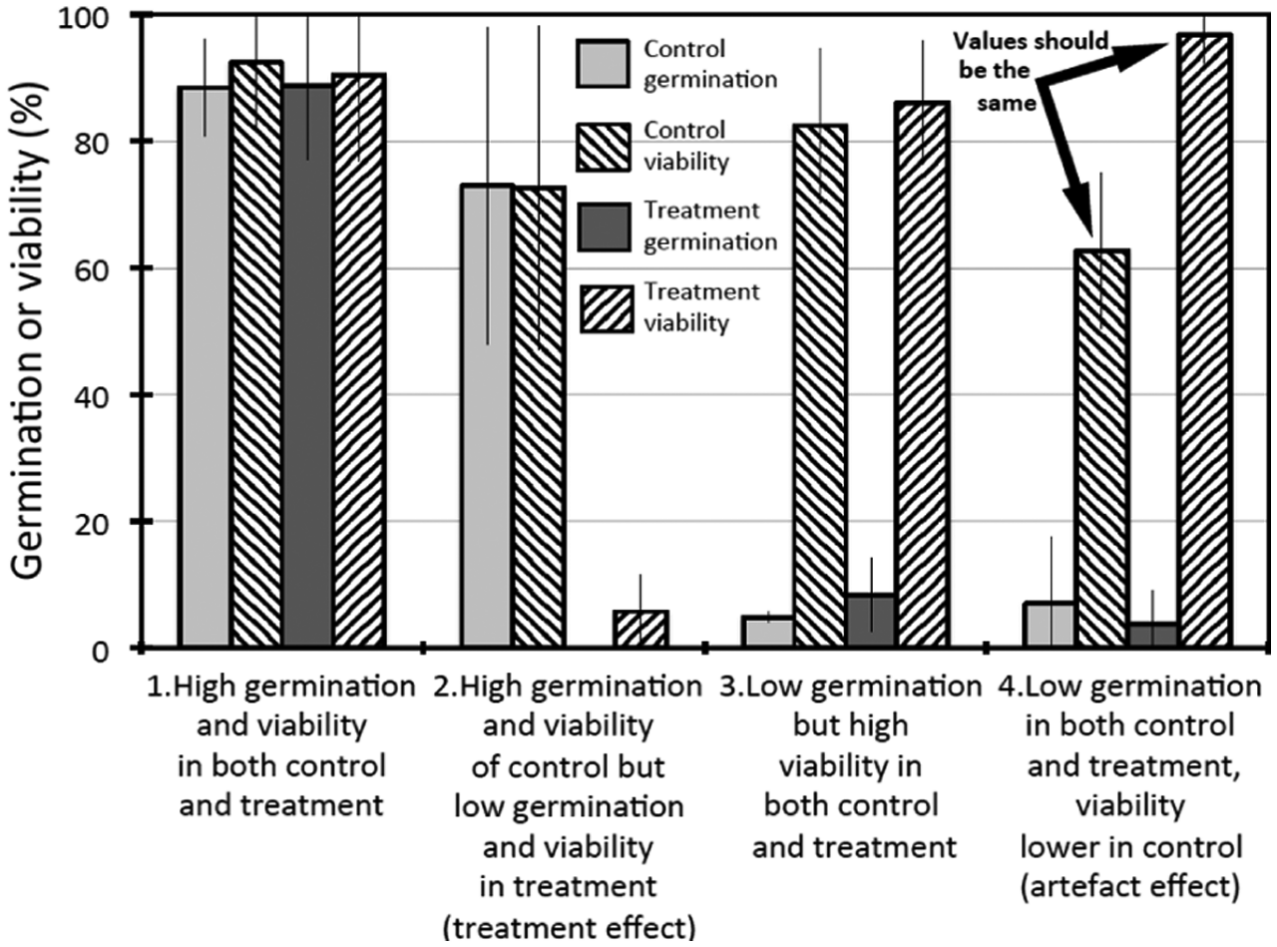
Example of anomalous reduction in estimated viability among some controls. I divided the data of Gómez‐González *et al*. (2017) into four categories, based on varying germination and viability pairings, determined at the end of the trial, with three species in each category. Results are means ± 95% CI. Note the last category where viability of the control is on average 36% less than the heat treatment (100 °C for 3 min), which must be an artefact of the experimental method. Also note that the unexpectedly low control values occur among species with little germination among the controls, but this is not unique in that respect (for example, see category 3).

As an example of the potential problem, my collation of data from Gómez‐González *et al*. (2017) highlights a case where estimates of viability for three species were anomalously low among the controls compared with the treatments (Fig. 1). Here, the last of the four categories into which I allocated each of the 12 species showed that estimated initial viability of the control was on average 36% less than the heat treatment (100 °C for 3 min). This must be an artefact of the experimental method as, at best, viability of the two should be the same (as in categories 1 and 2) or, at worst, the treated seeds would have lower viability at the end of the trial than the controls if the heat was excessive, as in category 2 (not the reverse, as here). Note that the unexpected effect occurs among species with little germination of the controls (consistent with the observations for *Leucadendron* above), but it is not unique in that respect (for example, see category 3 that also has low percentage germination but without reduction in viability). Gómez‐González *et al*. (2017) did not correct their data for initial viability, so that this treatment artefact was neither recognized (certainly not noted) nor used in determining germination success of the various treatments. In addition, Fidelis et al. (2016) noted a mean fungal infection rate among controls of four legumes of 53.9% and viability of 29.6% but when receiving a 60′C heat pretreatment this was 44.9% and 35.9% respectively (*P* = 0.0158, 0.0696 by paired *t*‐test), indicating greater infection and loss of viability among the controls rather than the heat treatment. Final viability was also (possibly erroneously) used to estimate initial viability in this study.

**Fig. 2:**
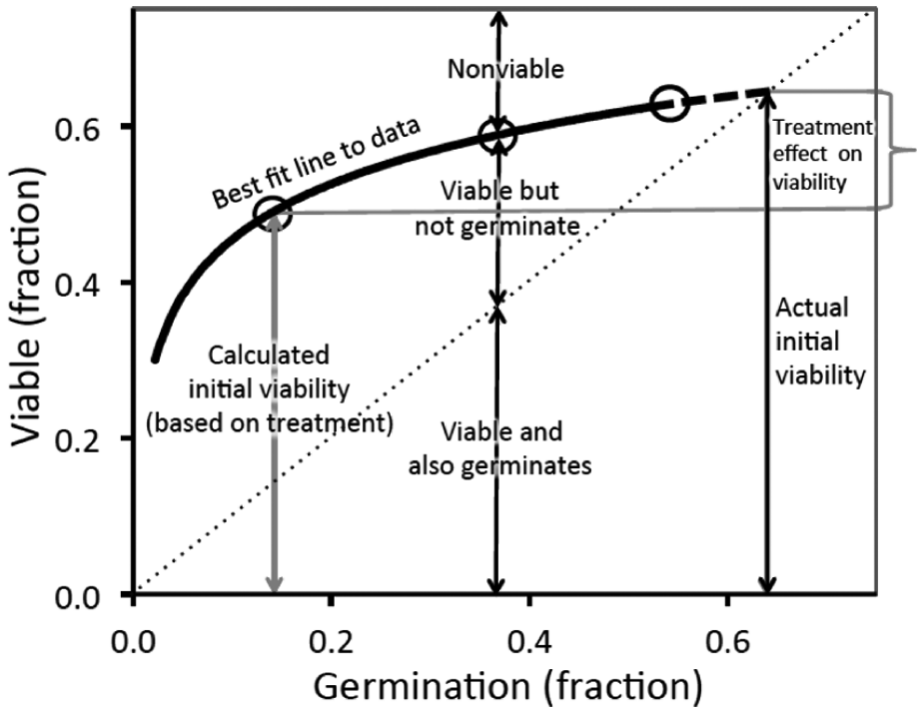
A hypothetical scenario where the viability of the seeds that remain ungerminated under various treatments, especially the control, lost viability during the trial (based on unpublished data of Newton *et al*. 2021). The circles correspond to (idealized) data points that fit on the best‐fit curve. The diagonal represents the situation where all seeds that were viable, germinated. The curve can be extrapolated back to the diagonal to provide an estimate of ‘true’ initial viability, *i.e*. viability at the start of the experiment, as required for estimating the success of the various treatments in breaking dormancy. The boxed area (treatment effect on viability) represents the extent of underestimation of true initial viability if, for example, it was based on the far‐left data point.

Based on the above findings, I have prepared Fig. 2 to show the type of pattern that can emerge when there is a treatment effect on seed viability. Here, the lower the final germination, the greater the probability of viability loss during the trial. Thus, values for initial viability become the dependent variable. If this problem was not recognized, and one of the treatments (or the mean of all treatments) was used to determine initial viability, then this value clearly underestimates ‘true’ initial viability.

Thus, end‐of‐trial assessment of initial viability can produce misleading estimates of germination success if it is based on (apparent) initial viability per treatment where this varies markedly between treatments. The reason is that the treatment itself, especially the control, may unexpectedly cause some seeds to die. Taking the approach of using germination as the independent variable and viability as the dependent variable, it is evident that the lower the germination rate, the more likely ungerminated seeds will lose viability during the trial (Fig. 2). Thus, it seems that the longer the seeds sit in the medium ungerminated, the more likely they will lose viability. That is, the control treatments, where there is little germination response, are most likely to lead to viability loss. A procedural problem is therefore indicated when the controls show lower estimated initial viability than some treatments (*e.g*. smoke treatment among some soil‐stored species in Hall *et al*. 2017; heat treatment of legume seeds in Gómez‐González *et al*. 2017; see Fig. 1).

## POSSIBLE SOLUTIONS

Since seed viability loss has a time‐dependent component (Ellis & Roberts 1980), if independent assessment of initial viability during the trial is not practicable, then there is a case for avoiding prolonged duration of ungerminated seeds in the treatments. But the answer is not to terminate the trial early, as species vary greatly in their rates of germination; use some other rule, such as terminate if no further germination at twice the interval since the last germination was recorded. Note that terminating the trial at different times between treatments is not a problem as the objective is for all treatments to reach the same stage (start of asymptote) rather than choosing an arbitrary common time when this stage may not have been reached among some slow‐germinating treatments.

In addition, the best‐fit line can be extrapolated back to the diagonal (*X* = *Y*; Fig. 2) and this value (estimated *Y*) can be used for initial viability in calculating germination success. For example, if a linear fit is used, then *Y* = *a*/(1 – *b*), where *a* is a constant and *b* is the slope. For a power function fit, log *Y* = log *a*/(1 – *b*). Note that this point may be close to 100%, independent of treatment effects on viability. Probability terms (*e.g*. confidence intervals) can also be added to the means. If the figure is substantially <100%, whether there is a negligible effect of treatment on the level of estimated viability or not, then either (i) this represents low viability at the start, or (ii) the general experimental design has led to a loss of viability. If a wide range of related species is used and most values approach 100%, the latter possibility seems unlikely. Where no trend line can be detected, other approaches are required. As one less satisfactory compromise, the treatment that gives the highest viability estimate can be used and applied to all treatments and control (Clarke *et al*. 2000), which is often near the extrapolated viability value anyway (Fig. 2). At least the problem is not ignored, as it is currently.

## CONCLUSIONS

As a result of this short review, I caution against the routine use of end‐of‐trial assessment of initial viability in determining germination success of various treatments. Values may prove to have been affected by the experimental design. This could be particularly serious where a wide range of treatments may have vastly different effects on estimated viability. The problem may be especially difficult to detect when in fact some treatments, *e.g*. application of fire‐type heat, can be expected to cause loss of viability.

The preference is for viability estimates to be undertaken on a separate sample of seeds just before the experimental trial begins, or concurrently with the trial. This is especially important where pretreatments, *e.g*. high temperatures, are expected to kill some seeds (Liyanage & Ooi 2017). Here, of course, seed viability at the end of the trial is used to determine if in fact the treatment has affected viability relative to initial viability that clearly must be determined independently. This means that, to minimize sample effects on pretrial estimates of initial viability, the number of seeds tested needs to be at least equivalent to the number used in the various treatments. Of course, identifying empty (no embryo), damaged or infested seeds at all stages of the trial is required (Leonard *et al*. 2018), as a separate task from pretrial determination of initial viability of intact seeds.

Note that there may be merit in determining the most suitable method for estimating viability before the experiment begins as a separate issue. Thus, Lamont & van Leeuwen (1988) showed that there was no difference in estimates of viability of *Banksia tricuspis* using the cut and tetrazolium tests, and thus opted for routine testing with the simpler cut test. It has not been my purpose here to compare different methods of determining seed viability (of which there are many; see Table 1), but I note that sometimes results are based on germinable (imbibed) seeds rather than checking directly for viability (Herranz *et al*. 1999). However, even imbibed seeds may be nonviable (dead tissues can imbibe), a problem that is exacerbated when there are treatment effects on viability, as described here.

It is a relevant final point to consider whether end‐of‐trial viability should also be determined routinely to assess if there is an overall experimental design effect (Liyanage & Ooi 2017). This is important, as the ability of the treatment to break dormancy is usually gauged *via* the level of germination. If final viability is low and treatment independent, it raises the issue of whether the general germination conditions imposed are unsuitable for that species, and that other design approaches should be considered. Possibilities include using different germination media, such as washed sand; different incubation temperature regimes; different light and dark exposures; and methods that avoid anoxia due to waterlogging. The method of determining viability may also underestimate levels, *e.g*. meristematic tissues need to be active for responses to the tetrazolium test (Gosling 2003).
